# Mir-615-5p inhibits cervical cancer progression by targeting TMIGD2

**DOI:** 10.1186/s41065-024-00363-7

**Published:** 2025-01-09

**Authors:** Yan Zhao, Haitao Chen, Wenhui Zhang, Wei Shang, Jinwei Cao, Huijuan Zhao, Zhixiang Zou

**Affiliations:** 1 Department of Gynecology, Taiyuan Central Hospital Affiliated to Shanxi Medical University, Taiyuan, Shanxi China; 2 Department of Obstetrics and Gynecology, Zhucheng Hospital of Traditional Chinese Medicine, Weifang, 262200 Shandong China; 3 Department of Medical Imaging, The Third Hospital of Shijiazhuang, No. 15, Tiyu South Street, Chang’an District, Shijiazhuang, 050051 Hebei China; 4 Department of Obstetrics and Gynecology, Renqiu People’s Hospital, Renqiu, 062550 Hebei China; 5 Department of Medical Imaging, The Sixth Hospital of Shijiazhuang, Shijiazhuang, 050051 Hebei China; 6 Second Department of Obstetrics and Gynecology, The Sixth Hospital of Shijiazhuang, Shijiazhuang, 050051 Hebei China; 7 Obstetrics and Gynecology Medical Centre, The First Affiliated Hospital of Hunan University of Traditional Chinese Medicine, No.105, Shaoshan Middle Road, Yuhua District, Changsha, 410007 Hunan China

**Keywords:** miR-615-5p, Cervical cancer, Prognosis, Progression, TMIGD2

## Abstract

**Background:**

Cervical cancer (CC) is a prevalent gynecological malignancy, contributing to a substantial number of fatalities among women. MicroRNAs (miRNAs) have emerged as promising biomarkers with significant potential for the early detection and prognosis of CC.

**Objective:**

This study aimed to explore the clinical significance and biological role of miR-615-5p in CC, with the goal of identifying novel biomarkers for this disease.

**Materials and methods:**

The levels of miR-615-5p and TMIGD2 mRNA in tissue samples and cells were quantified through quantitative reverse transcription real-time PCR, followed by statistical analyses to investigate the correlation between miR-615-5p and clinical data. The effects of miR-615-5p on the proliferation and metastasis of CC cells were evaluated using the Cell Counting Kit-8 and Transwell assays. The potential mechanism of miR-615-5p was elucidated by bioinformatics analyses and Dual-luciferase reporter assay. Western blotting was employed to measure the protein levels of TMIGD2.

**Results:**

In CC, the downregulation of miR-615-5p was related to poor prognosis and emerged as an independent prognostic factor. The levels of miR-615-5p were reduced in CC cells. miR-615-5p overexpression restrained the proliferation and metastasis of CC cells. Furthermore, TMIGD2 was identified as a target gene regulated by miR-615-5p, and its expression was notably elevated in CC. The influence of miR-615-5p on the biological behaviors of CC cells was mediated through the modulation of TMIGD2.

**Conclusions:**

Downregulation of miR-615-5p was a prognostic indicator of poor prognosis in CC. miR-615-5p exerted its tumor-suppressive effects by inhibiting cell growth and metastasis through the regulation of TMIGD2.

**Supplementary Information:**

The online version contains supplementary material available at 10.1186/s41065-024-00363-7.

## Background

Cervical cancer (CC) is a malignancy that poses a significant threat to women’s health worldwide, ranking as the fourth most common cancer in terms of both incidence and mortality among women [1]. The primary etiological factor for CC is persistent infection with high-risk human papillomavirus (HR-HPV), particularly HPV types 16 and 18, which are responsible for approximately 70% of all CC cases [2]. Early detection through CC screening is a critical strategy for identifying preclinical or early-stage CC, offering patients the opportunity to undergo timely and standardized treatment, thereby improving their prognosis [3, 4]. However, due to limited awareness of the importance of screening, many women are diagnosed at advanced stages, often with metastatic disease, missing the optimal window for intervention. As a result, their 5-year survival rates are significantly diminished [5]. For patients diagnosed with early-stage CC, surgery remains the treatment of choice, providing not only the opportunity to remove the primary tumor but also enabling the evaluation of tumor infiltration and metastasis. This assessment is essential for selecting appropriate adjuvant therapies and for accurate prognostic evaluation [6]. Consequently, identifying novel tumor markers and therapeutic targets holds great promise for enhancing early diagnosis and treatment, while also playing a pivotal role in reducing the risk of recurrence, thus offering significant clinical benefits in managing the disease.

Studies have substantiated that microRNAs (miRNAs) play a critical role in negatively regulating the expression of target genes, thereby influencing various cellular biological processes [7]. It has been demonstrated that miRNAs are intricately associated with the onset of numerous cancers, actively participating in pivotal mechanisms of cancer development, including proliferation, apoptosis, angiogenesis, energy metabolism, and immune evasion [8, 9]. Recent investigations have shed light on the aberrant expression of miRNAs in CC and their contributions to tumor progression [10]. For instance, dysregulated miR-122 has been implicated in prognosis related to CC and is known to influence the malignant progression through its regulation of RAD21 [11]. Additionally, research conducted by Guan et al. discovered that miR-615-5p plays a significant role in ovarian cancer progression and is intricately linked to tumor metastasis [12]. Xu et al. elucidated that miR-615-5p exhibited differential expression in response to the overexpression of HPV16 oncoprotein E6, as highlighted by microarray analysis [13]. Furthermore, a recent study has identified that miR-615-5p functions as a target miRNA of circ_0006789 in CC [14]. Therefore, miR-615-5p was hypothesized to be a promising prognostic biomarker and therapeutic target in CC. The clinical implications of miR-615-5p in CC, along with the underlying downstream mechanisms, warrant further exploration and elucidation.

The current study underscores the clinical relevance of miR-615-5p in CC and delves into its role in the progression of the disease, with the aim of offering valuable insights to inform clinical treatment strategies for CC.

## Methods

### Study subjects

A total of 131 patients diagnosed with CC who underwent surgical treatment at The Third Hospital of Shijiazhuang between January 2017 and January 2019 were included in this study. The research was approved by the Ethics Committee of the hospital and conducted in accordance with the principles of the Declaration of Helsinki. Inclusion criteria: (1) Patients were diagnosed by qualified specialists and confirmed by postoperative pathological examination. (2) All cases were primary occurrences, with complete and accessible pathological data. (3) Patients had not received any radiotherapy, chemotherapy, or other treatments prior to surgery. Exclusion criteria: (1) Co-existing malignancies or serious infectious diseases; (2) Inability to complete the follow-up. All participants provided informed consent prior to inclusion in the study. Pathological data were meticulously compiled, and tissue samples—comprising both cancerous and adjacent normal tissues—were collected from each patient. A five-year follow-up was completed for all subjects.

### Total RNA extraction

Tissue samples were homogenized using ultrasonication, and RNA was subsequently extracted with TRI Reagent (Sigma-Aldrich, USA). The RNA concentration was then quantified using a NanoDrop 2000 spectrophotometer (Thermo, USA). For cell RNA extraction, TRI reagent was directly added to the cell samples, and the remaining procedures were performed according to the manufacturer’s instructions.

### qRT-PCR

PrimeScript RT reagent Kit (Takara, Japan) was employed for the reverse transcription of TMIGD2. For miR-615-5p reverse transcription, Mir-X miRNA First-Strand Synthesis Kit (Takara) was utilized, incorporating poly(A) polymerase. The relative levels of miR-615-5p and TMIGD2 mRNA in the tissues were quantified using the CFX Connect RT-qPCR system (Bio-Rad, USA) and SYBR Green qPCR Master Mix (Applied Biosystems, USA). Data were analyzed using the 2^−ΔΔCt^ method. U6 (for miR⁃615⁃5p) and β-actin (for TMIGD2 mRNA) served as internal controls. The primers used are shown in Supplementary Material (Table S1)

### Western blot

The transfected cells were harvested and lysed with RIPA lysis buffer, followed by incubation on ice for 30 min to ensure complete cell lysis. Protein extraction was achieved by centrifugation at 12,000 rpm at 4 °C for 20 min. The resulting samples were then boiled for 5 min to ensure complete denaturation of the proteins. SDS-PAGE was conducted to separate the proteins, after which the proteins were transferred onto the PVDF membrane. The membrane was blocked with 5% skimmed milk for 1 h to prevent non-specific binding. Primary antibodies against TMIGD2 (H00126259-B01P, Abnova, USA) and β-actin (ab115777, Abcam, UK) were incubated with the membrane overnight at 4 °C. The membrane was subsequently incubated with HRP-conjugated goat anti-rabbit secondary antibody (ab205718, Abcam) for 1 h. After each incubation step, the membrane was washed three times with PBST to remove unbound antibodies. Protein signals were detected using ECL chemiluminescence, and the band intensity was quantified using ImageJ software, with β-actin serving as the loading control for normalization.

### Cell lines

CC cell lines (HeLa, SiHa, C-3AA, CaSki) and normal human cervical epithelial cells (HCerEpiC) were all procured from the Shanghai Cell Bank (Shanghai, China). HeLa, SiHa, and C-3AA cells were cultured in MEM medium, while CaSki cells were maintained in RPMI 1640 medium. All media was supplemented with 1% penicillin/streptomycin and 10% fetal bovine serum (FBS) (Gibco, USA). Cells were incubated at 37 °C in a humidified atmosphere containing 5% CO_2_.

### Transfection

The pcDNA3.1 vector (Thermo) was employed to construct the TMIGD2 overexpression plasmid, designated as pcDNA3.1-TMIGD2. To modulate the expression of miR-615-5p, either miR-615-5p mimic (GGGGGUCCCCGGUGCUCGGAUC) or inhibitor (GAUCCGAGCACCGGGGACCC) (MedChemExpress, USA) was employed, with their respective negative controls being mimic NC (UUGUACUACACAAAAGUACUG) and inhibitor NC (CAGUACUUUUGUGUAGUACAA). Cell transfection was performed following the manufacturer’s protocol using Lipofectamine 3000 Transfection Reagent (Thermo).

### CCK-8 assay

The transfected cells were harvested to prepare suspensions, followed by cell counting. Cell suspension (100 µL/well) was inoculated in a 96-well plate, ensuring five replicate wells per treatment. The cells were maintained in culture, and the medium was supplemented with 10% CCK-8 reagent (Dojindo, Japan) as a replacement. CCK-8 reagent was added at 0, 24, 48, and 72 h, with the experiment being conducted in triplicate. After the addition of the reagent, the cells continued to culture for 1 h, after which cell proliferation was assessed by measuring optical density at 450 nm (OD_450_) using an enzyme labeler.

### Transwell assay

Post-transfected cells were harvested and resuspended in serum-free medium. The cells were subsequently seeded onto the upper chamber of the Transwell, while the lower chamber was filled with cell culture medium. After 24 h of incubation, the medium was carefully aspirated. The cells were then fixed with 4% paraformaldehyde and stained with 0.1% crystal violet solution. Migrated cells were visualized using the DMi1 inverted microscope (Leica, Germany) and photographed for analysis. For the invasion assay, Matrigel Matrix (Corning, USA) was diluted with ice-cold serum-free medium and applied evenly to the surface of the Transwell membrane in the upper chamber. The Matrigel was allowed to polymerize at 37 °C for 3 h, forming a gel layer. Following this incubation, the excess liquid was removed, and the basement membrane was rehydrated by adding serum-free medium, followed by a 30-minute incubation at 37 °C. The remaining steps were carried out as previously described.

### Bioinformatics analysis

TMIGD2 expression was evaluated using the online UALCAN database (https://ualcan.path.uab.edu/analysis.html). Predicted target genes were identified and screened through the online databases TargetScanHuman (https://www.targetscan.org/vert_72/), miRWalk (http://mirwalk.umm.uni-heidelberg.de/), and miRDB (https://mirdb.org/mirdb/index.html). The intersection of the predicted target genes was obtained and visualized via a Venn diagram. Additionally, the TargetScanHuman database was utilized to predict potential binding sites for the target genes.

### Dual-luciferase reporter assay

The pGL3 vector (Promega, USA) was employed to construct TMIGD2 luciferase reporter plasmids which included either the wild-type binding sites for miR-615-5p (TMIGD2-WT) or the corresponding mutant binding sites (TMIGD2-MUT). HeLa and SiHa cells were seeded in 24-well plates and subsequently transfected with pGL3-TMIGD2-WT or pGL3-TMIGD2-MUT plasmids, in combination with miR-615-5p mimic, inhibitor, or negative control, according to the experimental design. After 48 h of incubation, cell lysates were harvested and analyzed using the Dual-luciferase Reporter Assay System (Promega) with the Varioskan LUX multifunctional plate reader (Thermo). The relative luciferase activity was then calculated based on the recorded data.

### Statistical analysis

All statistical analyses and graphing were conducted using SPSS 23.0 and GraphPad Prism 9.3. Group comparisons were performed using Student’s t-test or one-way ANOVA as appropriate. The association between miR-615-5p and pathological data was examined using the Chi-square test. The prognostic significance of miR-615-5p in CC was assessed through Kaplan-Meier curve and Cox regression analysis. The relationship between miR-615-5p and TMIGD2 was evaluated using Pearson’s correlation. A *p*-value of < 0.05 was considered statistically significant.

## Results

### Expression and clinical significance of miR-615-5p in CC

**Fig. 1:**
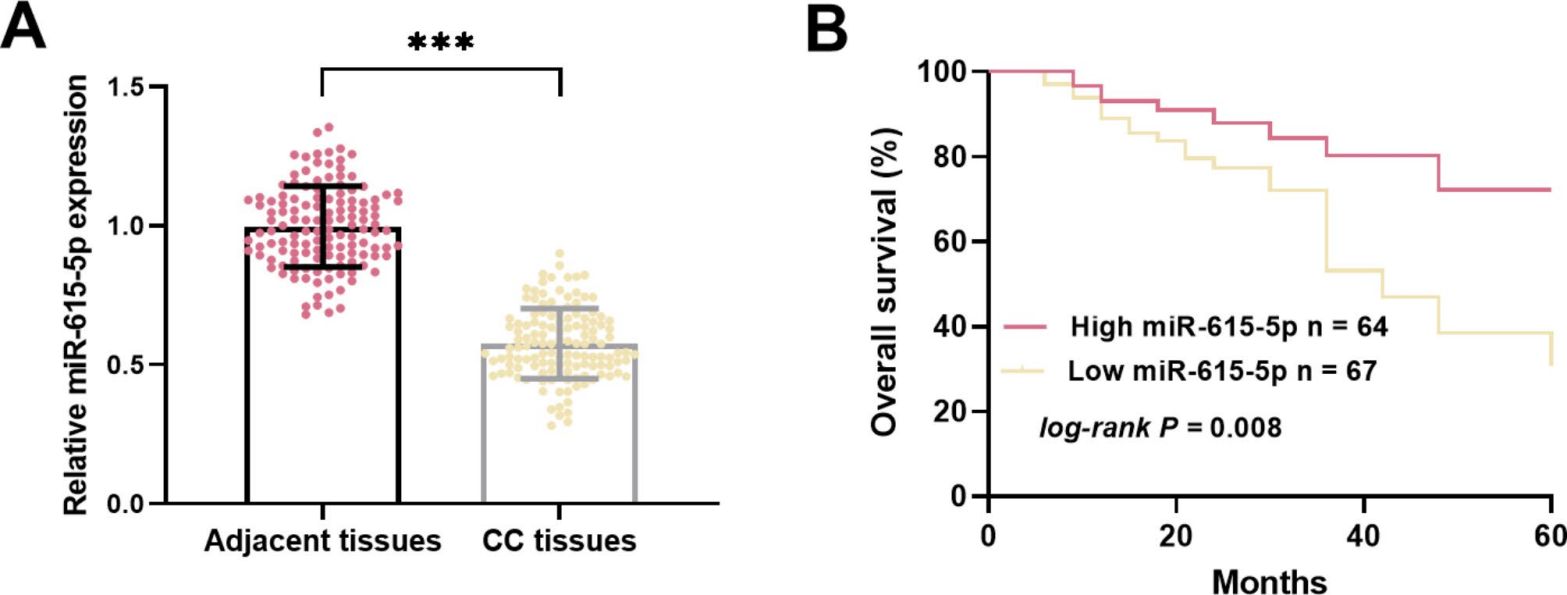
Downregulation of miR-615-5p in CC and its prognostic implications. **(A)** Decreased expression of miR-615-5p in CC tissues. **(B)** Low expression of miR-615-5p correlates with poor prognosis in CC patients. ***P < 0.001

A significant decrease in the levels of miR-615-5p was observed in CC tissues compared to adjacent normal tissues (Fig. 1A). Based on the average miR-615-5p level (0.575) in all CC tissues, patients were stratified into two groups: high-miR-615-5p (*n* = 64) and low-miR-615-5p (*n* = 67). miR-615-5p expression was found to be significantly associated with FIGO stage (*P* = 0.008) and lymph node metastasis (*P* = 0.018) (Table 1). Survival analyses revealed that patients with higher miR-615-5p levels exhibited significantly better survival outcomes (*log-rank*, *P* = 0.008, Fig. 1B). Furthermore, miR-615-5p (HR = 2.717, 95% CI = 1.107–6.667, *P* = 0.029), lymph node metastasis (HR = 2.650, 95% CI = 1.093–6.423, *P* = 0.031) and FIGO stage (HR = 3.669, 95% CI = 1.140-11.805, *P* = 0.029) were identified as independent prognostic factors (Table 2).

**Table 1:** Correlation of mir-615-5p and TMIGD2 expression with clinical characteristics of CC patients

Characteristic	Cases (*n* = 131)	miR-615-5p level		*P* value	TMIGD2 level		*P* value
		Low (*n* = 67)	High (*n* = 64)		Low (*n* = 66)	High (*n* = 65)	
Age (years)				0.140			0.254
≤ 45	63	28	35		35	28	
> 45	68	39	29		31	37	
Histological type				0.448			0.545
Squamous carcinoma	74	40	34		39	35	
Adenocarcinoma	57	27	30		27	30	
Tumor size (cm)				0.064			0.434
≤ 4	69	30	39		37	32	
> 4	62	37	25		29	33	
Lymph node metastasis				0.018			0.009
No	85	37	48		50	35	
Yes	46	30	16		16	30	
FIGO stage				0.008			0.010
I-II	79	33	46		47	32	
III	52	34	18		19	33	
Differentiation				0.081			0.187
Well/moderate	78	35	43		43	35	
Poor	53	32	21		23	30	
LVSI				0.549			0.434
No	62	30	32		29	33	
Yes	69	37	32		37	32	

Abbreviations: CC, cervical cancer; FIGO, International Federation of Gynecology and Obstetrics; LVSI, lymph vascular space invasion

**Table 2:** Cox regression analysis of clinical characteristics of CC patients

Characteristic	HR	95% CI	*P* value
miR-615-5p	2.717	1.107–6.667	0.029
Age	1.352	0.610–2.998	0.457
Histological type	1.484	0.647-3.400	0.351
Tumor size	2.094	0.908–4.826	0.083
Lymph node metastasis	2.650	1.093–6.423	0.031
FIGO stage	3.669	1.140-11.805	0.029
Differentiation	1.605	0.741–3.477	0.230
LVSI	1.922	0.878–4.205	0.102

Abbreviations: CC, cervical cancer; FIGO, International Federation of Gynecology and Obstetrics; LVSI, lymph vascular space invasion; HR, hazard ratio; CI, confidence interval

### Mir-615-5p inhibited the biological behaviors of CC cells

**Fig. 2:**
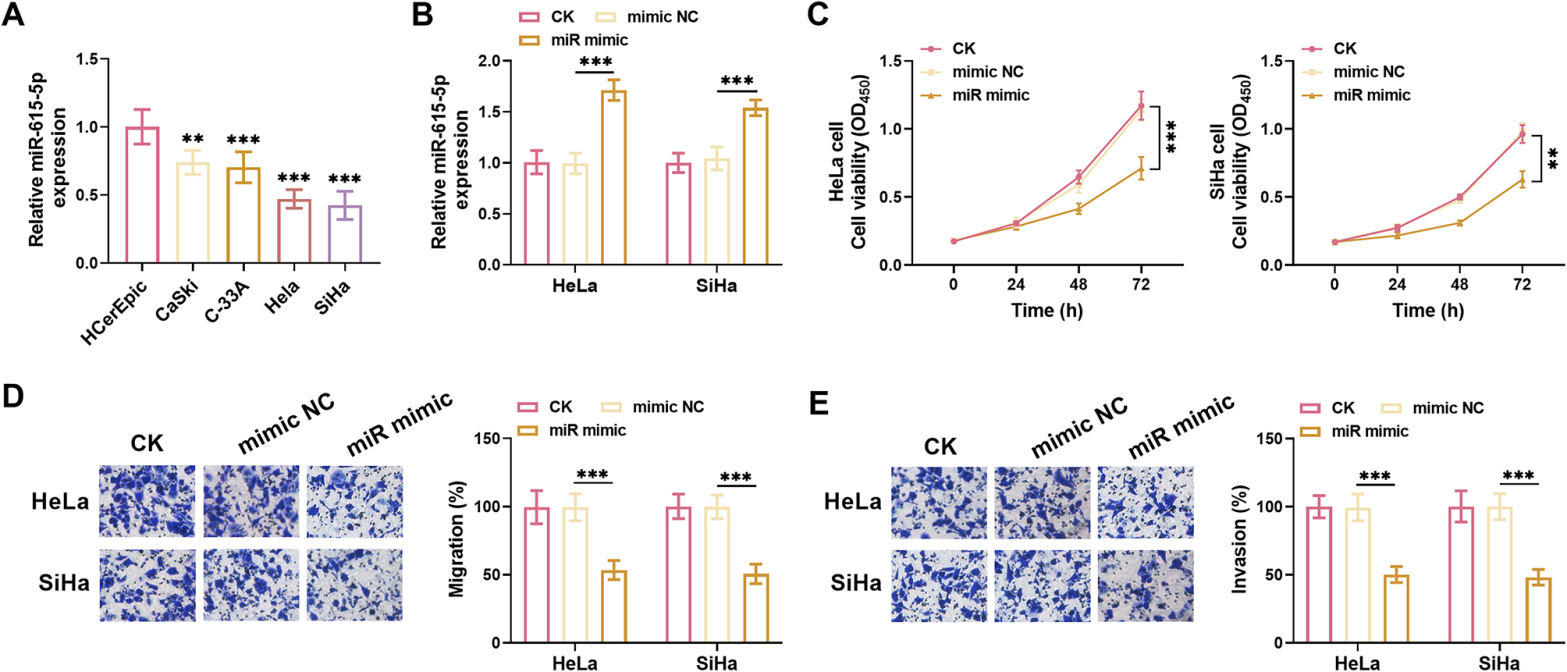
The inhibitory effects of miR-615-5p on the biological functions of CC cells. **(A)** The level of miR-615-5p was found to be significantly lower in CC cells (HeLa, SiHa, C-3AA, CaSki) compared to normal cervical epithelial cells (HCerEpiC). **(B)** The levels of miR-615-5p were upregulated by miR-615-5p mimic. **(C)** The proliferation of HeLa and SiHa cells was notably suppressed following the upregulation of miR-615-5p. **D**,** E.** miR-615-5p significantly inhibited both the migration (**D**) and invasion (**E**) of HeLa and SiHa cells. **P < 0.01, ***P < 0.001

miR-615-5p expression was dramatically reduced in CC cells compared to HCerEpic cells, particularly in HeLa and SiHa cells (Fig. 2A). Transfection with the miR-615-5p mimic led to a substantial increase in miR-615-5p expression, surpassing the levels observed in control cells (Fig. 2B). Notably, miR-615-5p mimic markedly restrained the proliferation, migration, and invasion of both HeLa and SiHa cells (Fig. 2C-E). Furthermore, cells transfected with miR-615-5p mimic exhibited a prolonged doubling time (Supplementary Material, Figure S1-A).

### TMIGD2 was a target gene of miR-615-5p

**Fig. 3:**
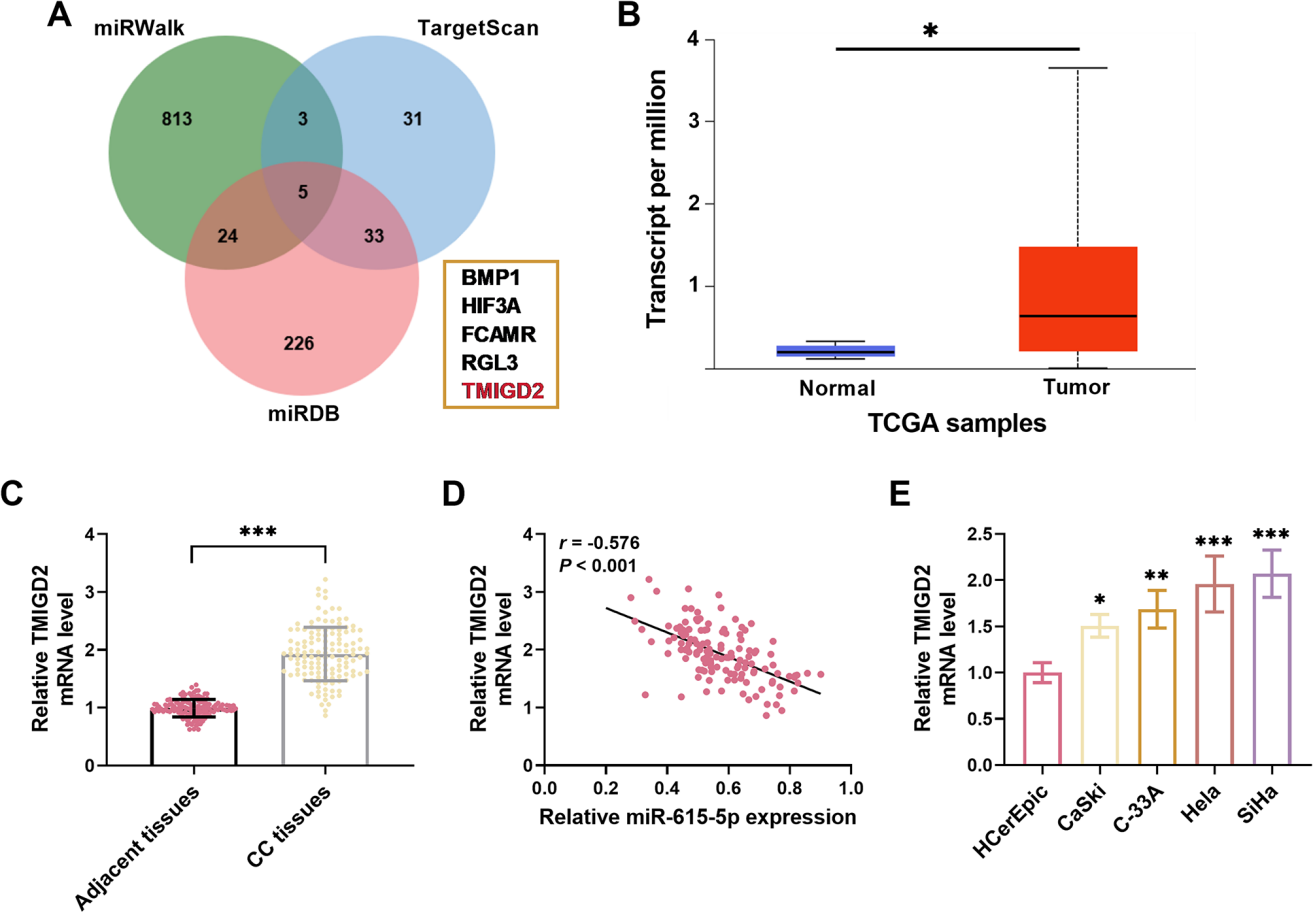
miR-615-5p targeted TMIGD2 in CC. **(A)** A total of five potential target genes for miR-615-5p were identified using the miRWalk, TargetScanHuman, and miRDB databases. **(B)** Data from the UALCAN database revealed elevated mRNA levels of TMIGD2 in CC tissues. **(C)** An increase in TMIGD2 mRNA expression was observed in CC tissues compared to normal controls. **(D)** A negative correlation was detected between miR-615-5p levels and TMIGD2 mRNA expression in CC tissues. **(E)** TMIGD2 mRNA levels were notably higher in CC cells compared to normal cervical epithelial cells. *P < 0.05, **P < 0.01, ***P < 0.001

The potential target genes of miR-615-5p were systematically predicted and screened utilizing the miRWalk, TargetScanHuman, and miRDB databases. Analysis via a Venn diagram revealed a total of five intersecting target genes, among which only the expression of TMIGD2 mRNA was significantly influenced by miR-615-5p in CC cells (Supplementary Material, Figure S2). Consequently, TMIGD2 was selected for further validation (Fig. 3A). Insights drawn from the UALCAN database indicated an elevation in TMIGD2 mRNA levels in CC (Fig. 3B). This upregulation of TMIGD2 mRNA was corroborated by analyses conducted on CC tissues included in this study (Fig. 3C). Moreover, a notable association was established between TMIGD2 mRNA levels and both lymph node metastasis (*P* = 0.009) and FIGO stage (*P* = 0.010, Table 1). Importantly, a negative correlation was observed between miR-615-5p and TMIGD2 mRNA levels (*r* = -0.576, Fig. 3D). Additionally, TMIGD2 mRNA levels exhibited a significant elevation in CC cells (Fig. 3E).

**Fig. 4:**
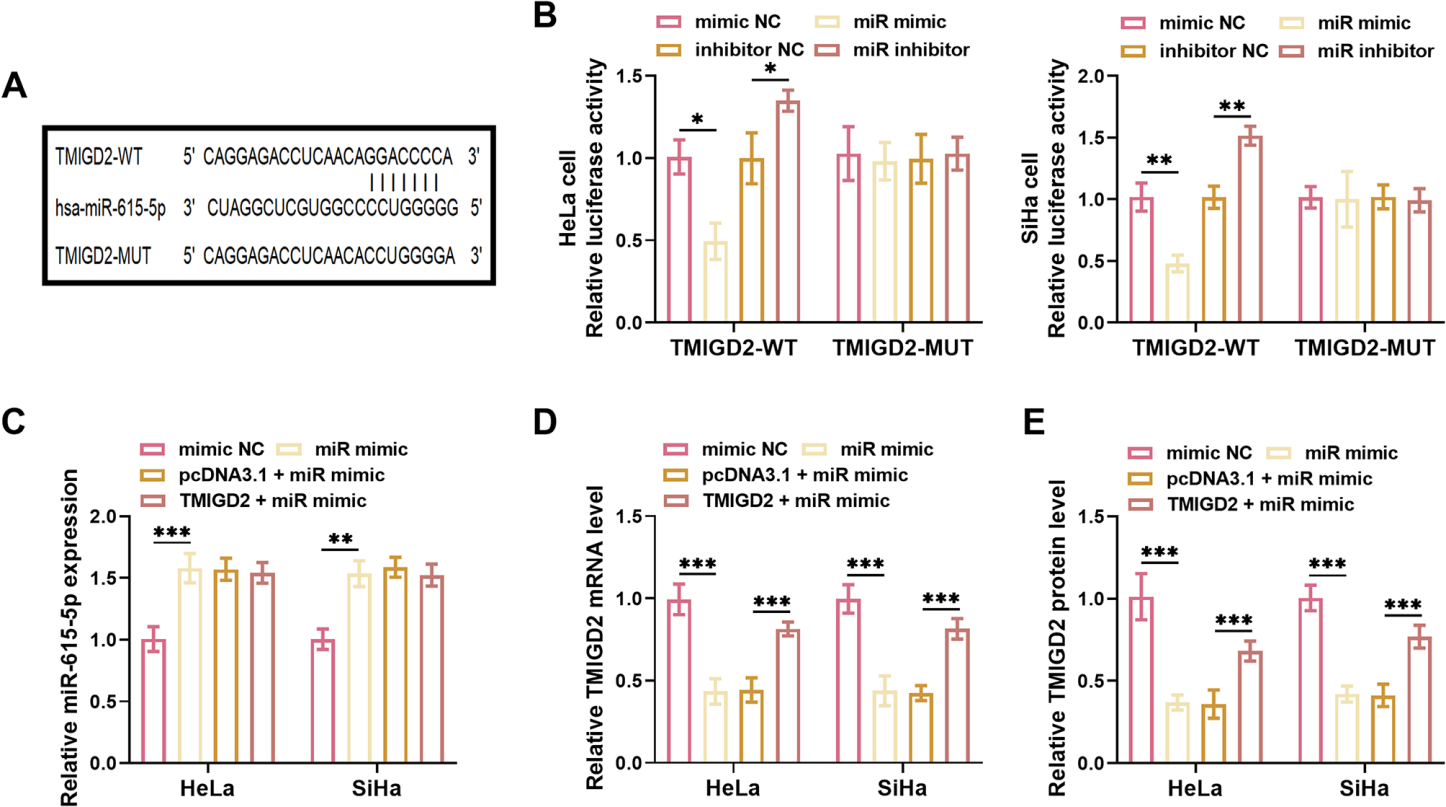
miR-615-5p negatively regulated TMIGD2 in CC. **(A)** TargetScanHuman database identified potential binding sites of TMIGD2 for miR-615-5p. **(B)** In HeLa and SiHa cells, upregulation of miR-615-5p led to a significant reduction in the luciferase activity of TMIGD2-WT, while its downregulation exhibited the converse effect. **(C)** The levels of miR-615-5p were dramatically enhanced by miR-615-5p mimic. **D**,** E.** Elevated expression of miR-615-5p resulted in the inhibition of TMIGD2 mRNA (**D**) and protein (**E**) expression, whereas the TMIGD2 overexpression vector effectively restored TMIGD2 levels. *P < 0.05, **P < 0.01, ***P < 0.001

The potential binding sites of miR-615-5p to TMIGD2 were predicted using the TargetScanHuman database (Fig. 4A). To further validate the interaction between TMIGD2 and miR-615-5p, luciferase reporter assays were performed. miR-615-5p significantly diminished the relative luciferase activity of SiHa and HeLa cells transfected with TMIGD2-WT, while miR-615-5p inhibitor enhanced the relative luciferase activity in these same cells. In contrast, no substantial changes in luciferase activity were observed in SiHa and HeLa cells transfected with TMIGD2-MUT, regardless of the presence of the miR-615-5p mimic or inhibitor (Fig. 4B). To further investigate the regulatory effect of miR-615-5p on TMIGD2 expression in vitro, co-transfection experiments were performed. The miR-615-5p mimic markedly increased the expression of miR-615-5p (Fig. 4C). Notably, overexpression of miR-615-5p resulted in a significant decrease in both the mRNA and protein levels of TMIGD2. However, this reduction was reversed by the introduction of the TMIGD2 overexpression vector (Fig. 4D, E).

### Mir-615-5p regulated CC cell biological behaviors via TMIGD2

**Fig. 5:**
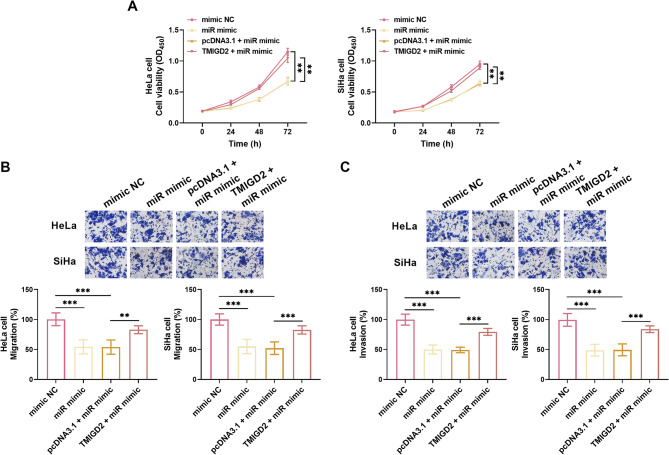
miR-615-5p modulates the malignant phenotype of CC cells through TMIGD2 regulation. **A.** miR-615-5p significantly inhibited the proliferation of both HeLa and SiHa cells, and this effect was reversed upon the upregulation of TMIGD2. **B-C.** Overexpression of miR-615-5p led to a marked reduction in the migration (**B**) and invasion (**C**) of HeLa and SiHa cells, and this effect was significantly attenuated by the upregulation of TMIGD2. **P < 0.01, ***P < 0.001

The biological role of miR-615-5p in CC progression was further elucidated. Compared to the group transfected with the pcDNA3.1 vector, overexpression of TMIGD2 dramatically reversed the inhibitory effect of the miR-615-5p mimic on cell proliferation in HeLa and SiHa cells (Fig. 5A). Furthermore, the impact of miR-615-5p mimic on cell doubling time was mitigated by TMIGD2 overexpression (Supplementary Material, Figure S1-B). Additionally, the suppressive effects of miR-615-5p mimic on cell migration and invasion were effectively alleviated by the TMIGD2 overexpression vector (Fig. 5B, C).

## Discussion

In clinical practice, the prognosis of CC is predominantly determined by key pathological factors such as lymph node metastasis, tumor size, and the depth of stromal invasion [15]. However, recent advancements in technology have led to the identification of numerous biomarkers that offer significant insights into both the diagnosis and prognosis of CC [16]. Additionally, hematological markers and other underlying health conditions have been shown to influence the survival of CC patients [17, 18]. Notably, this study discovered that the downregulation of miR-615-5p in CC samples was related to a worse prognosis. Moreover, miR-615-5p has been found to significantly attenuate the malignant characteristics of CC cells, suggesting its potential role as a suppressor of CC progression.

In recent years, miRNAs have been identified as exhibiting aberrant expression across various malignant tumors, where they play significant roles in cancer progression, functioning either as facilitators or inhibitors [19, 20]. For instance, miR-532-5p has been implicated in influencing patient prognosis through its role in promoting lymph node metastasis in CC [21]. Additionally, miR-218-5p, miR-95-3p, and miR-589-3p have emerged as potential prognostic indicators for CC prognosis [22–24]. Zhou et al. reported a notable downregulation of miR-615-5p in CC, and the present study further establishes that this downregulation correlates significantly with both lymph node metastasis and FIGO stage, thus indicating a poor prognosis [14]. Furthermore, our findings indicate that miR-615-5p, FIGO stage, and lymph node metastasis serve as independent prognostic predictors for patient outcomes. Collectively, these findings suggest that miR-615-5p may hold promise as a valuable prognostic biomarker in CC.

The pivotal role of miRNAs in CC has been well-documented in recent research. For instance, Wang et al. demonstrated that miR-19-3p promotes both invasion and proliferation in CC cells [25]. Conversely, miR-214-3p, miR-195-3p, and miR-186-3p have been identified as inhibitors of CC progression [26–28]. Previous studies have shown that while miR-615-5p acts as a tumor promoter in hepatocellular carcinoma, it functions as a tumor suppressor in colorectal cancer [29, 30]. Here, miR-615-5p was found to be markedly downregulated in CC cells. In gain-of-function assays, miR-615-5p effectively inhibited CC cell proliferation and metastasis, thereby underscoring its potential as a crucial therapeutic target in the management of CC.

miR-615-5p has emerged as a pivotal regulator in the progression of various cancers, where it exerts its influence by modulating gene expression. In glioma, miR-615-5p played a significant role in tumor growth by targeting huntingtin-interacting protein-1 (HIP1) [31]. Moreover, miR-615-5p acted as a tumor suppressor in non-small cell lung cancer by regulating P21-Activated Kinase 1 (PAK1) [32]. This study further elucidated the mechanisms and targets of miR-615-5p in CC through bioinformatics analyses, which were employed to identify potential target genes. Among those identified, TMIGD2 was observed to be upregulated in CC and demonstrated an inverse correlation with miR-615-5p levels. It was shown that miR-615-5p negatively modulates TMIGD2, significantly decreasing its expression in CC. Furthermore, the inhibitory effects of miR-615-5p on CC cell growth and metastasis were reversed upon TMIGD2 overexpression. A previous report by Boulhen et al. showed aberrant expression of TMIGD2 in glioma cells and suggested it as a potential therapeutic target [33]. Recent research has also revealed aberrant expression of TMIGD2 in acute myeloid leukemia cells, underscoring its critical role in disease progression [34]. The results of this study suggest that one of the key mechanisms through which miR-615-5p exerts its suppressive effect is via the downregulation of TMIGD2. TMIGD2 has been shown to facilitate multicellular aggregation in colorectal cancer, with disruption of its adhesion function impairing both multicellular aggregation and tumor growth [35]. Furthermore, emerging studies have indicated that TMIGD2 plays a significant role in stimulating the proliferation and migration of ovarian cancer cells [36]. It is speculated that TMIGD2 may promote multicellular aggregation through its adhesion function, thereby influencing cell proliferation and metastasis. Given its involvement in multiple malignancies, including endometrial cancer and small cell lung cancer, TMIGD2 is increasingly recognized as a promising therapeutic target across various cancers [37, 38]. These collective findings suggest that TMIGD2 may serve as an invaluable target for the treatment of CC, providing a foundation for the development of personalized treatment strategies tailored to individual patient profiles.

Further attention should be directed toward the limitations inherent in this study. The sample size utilized was relatively small, underscoring the necessity of incorporating a larger cohort for the validation and generalization of the results. Moreover, animal models play a pivotal role in advancing disease research, often providing data of superior accuracy and reliability compared to in vitro studies. In vivo experiments are indispensable for investigating the molecular mechanisms underlying CC progression. Therefore, it is crucial to corroborate the findings of this study through animal models, such as xenograft models, to more effectively assess the impact of miR-615-5p on tumor progression in vivo.

## Conclusions

In conclusion, the downregulation of miR-615-5p in CC tissues is indicative of a poorer prognosis for patients. miR-615-5p demonstrates an inhibitory effect on the proliferation, migration, and invasion of CC cells through the negative regulation of TMIGD2. These findings suggest that miR-615-5p serves as a promising biomarker and therapeutic target for CC, offering valuable insights for future treatment strategies.

## Electronic supplementary material

Below is the link to the electronic supplementary material.

Supplementary Material 1

## Data Availability

All data generated or analyzed during this study are included in this article. Further enquiries can be directed to the corresponding author.

## Abbreviations

CC: Cervical cancer
miRNAs: MicroRNAs
HR-HPV: High-risk human papillomavirus
