# Supplementary material for: Mir-615-5p inhibits cervical cancer progression by targeting TMIGD2
Source: Hereditas. 2025 Jan 9;162:4. doi: 10.1186/s41065-024-00363-7 (PMC11715597; doi:10.1186/s41065-024-00363-7)
Supplement: Supplementary file 1 — Supplementary Material 1 [file 41065_2024_363_MOESM1_ESM.docx]

**Table S1.** The primer sequences for qRT-PCR.

| Primer | Sequences (5’–3’) |
| --- | --- |
| miR-615-5p | Forward: GCATTTAGCAGCGAGACAA |
|  | Reverse: AGCGACACGTGCGAATGTTCT |
| TMIGD2 | Forward: AGAACAGAAACCGGATCGCA |
|  | Reverse: GGCTGTTACCTGAGTCCCTT |


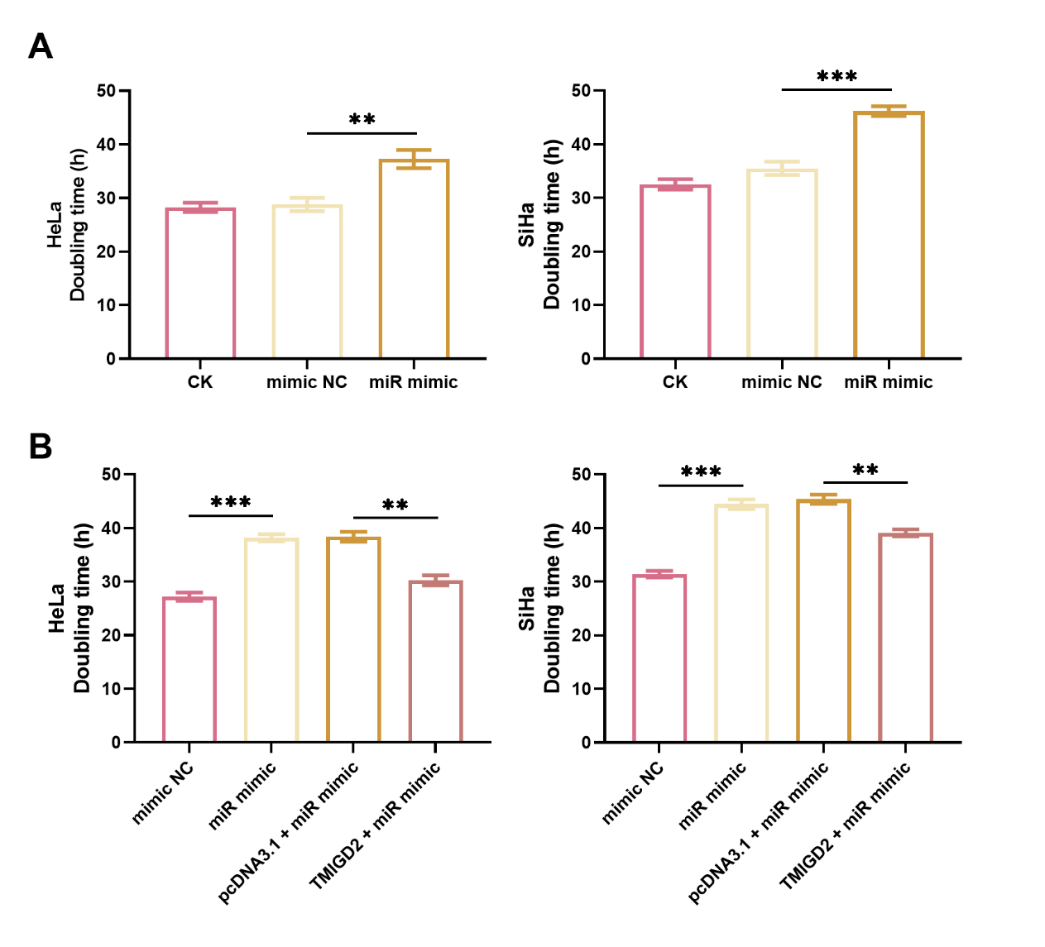


**Figure S1.** Cell doubling time in different treatment groups. **A.** The doubling time of cells transfected with miR-615-5p mimic was prolonged. **B.** The effect of miR-615-5p mimic on cell doubling time was attenuated by TMIGD2 overexpression.


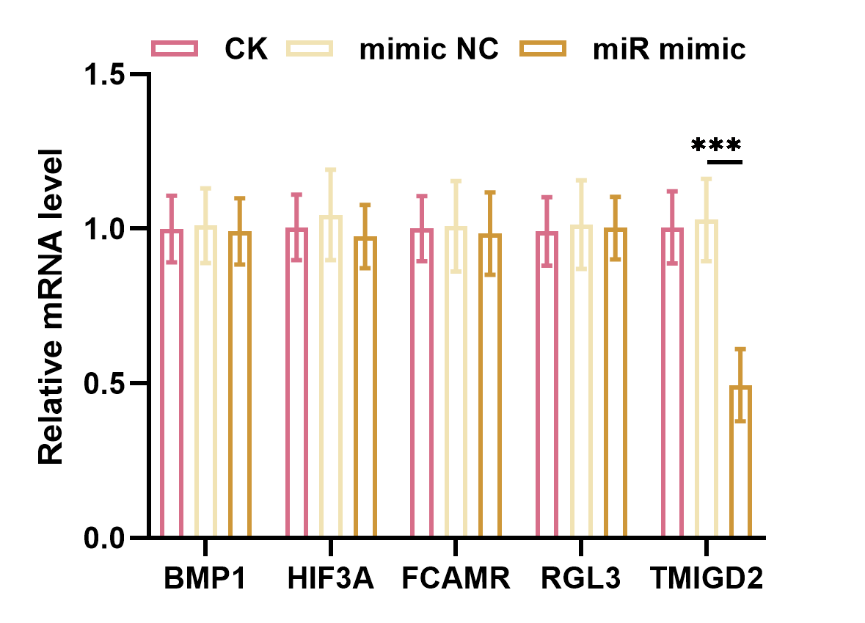


**Figure S2.** Effect of miR-615-5p on mRNA expression of potential target genes.
